# How photo editing in social media shapes self-perceived attractiveness and self-esteem via self-objectification and physical appearance comparisons

**DOI:** 10.1186/s40359-023-01143-0

**Published:** 2023-04-06

**Authors:** Phillip Ozimek, Semina Lainas, Hans-Werner Bierhoff, Elke Rohmann

**Affiliations:** 1 Mental Health Research and Treatment Center, Department of Psychology, Ruhr-Universität Bochum, Bochum, Germany; 2 Department of Social Psychology, Ruhr-Universität Bochum, Bochum, Germany

**Keywords:** Photo editing, Social media, Self-objectification, Physical appearance comparisons, attractiveness, Self-esteem

## Abstract

**Background:**

As photo editing behavior to enhance one?s appearance in photos becomes more and more prevalent on social network sites (SNSs), potential risks are increasingly discussed as well. The purpose of this study is to examine the relationship between photo editing behavior, self-objectification, physical appearance comparisons, self-perceived attractiveness, and self-esteem.

**Methods:**

403 participants completed self-report questionnaires measuring the aformentioned constructs. A parallel-sequential multiple mediation model was conducted to examine the relationship between photo editing behavior and self-esteem considering multiple mediators.

**Results:**

The results indicate that photo editing behavior is negatively related to self-perceived attractiveness and self-esteem mediated via self-objectification and physical appearance comparisons.

**Conclusions:**

The postulated mediation model was justified by our data. Thus, SNS users should be aware of potential negative consequences when using photo editing applications or filters.

**Supplementary Information:**

The online version contains supplementary material available at 10.1186/s40359-023-01143-0.

## Introduction

Sometimes I forget that I am human with a body, not a playdough that can be pressed and squeezed until it fits the predetermined mould this society has deemed “beautiful”.

- Anonymous

Social media represent digital platforms based on new communication technologies fostering new possibilities for carrying out social interaction and communication [1]. They are based on Web 2.0 technology which allows the sharing of information among a large number of persons. Social media provide computer-mediated communication channels enabling users to communicate with each other.

An example of social media are social network sites which are defined by the use of profiles, the embeddedness in networks, and by the use of streams [2]. Profiles contain personal attributes related to the users which enable them to present themselves positively. Therefore, users are not necessarily obliged to include only true information in their profiles. Instead, deceptive self-presentation is a viable alternative. An example is research on dating platforms showing that the information provided is not always sincere [3]. With respect to height, weight, and age, 81% of profiles were not accurate because underreporting (especially with respect to weight) and overreporting (especially with respect to height) occurred. In addition, profile photographs also were inaccurate to some extent. This was less the case the more friends and family members were aware if the online dating profile.

Obviously, temptations to whitewash the profile are weighted against reality anchors and issues of credibility. Another feature of social network sites is their embeddedness in smaller or larger networks of users. Furthermore, social network sites comprise user-generated messages which are encoded in streams.

Social media including social media sites constitute a new context of social interaction which is contrasted with face-to-face interaction and digital communication media like email and video conferencing [1]. Social media differ from face-to-face interaction and digital communication media with respect to a plethora of communication variables including accessibility, latency, physicality, interdependence, synchronicity, permanence, verifiability and anonymity resulting in the reduction of time and distance barriers. For example, physicality contrasts face-to-face interactions taking place in a material context, which is tangible and perceptible, with an artificial environment based on digitalization. In general, the artificial environment facilitates communication. In addition, latency is reduced on the internet because it takes less time to share content within the social network site in comparison to face-to-face communication. Furthermore, the truth of messages is easier to verify on social media sites (e.g., by background checks). In general, social media facilitate communication processes considerably by offering the sender more channels to share information with many recipients.

An important domain of profile information refers to the use of profile photographs, which are more or less accurate [3]. Photo editing behavior tends to improve the impression conveyed by the profile photo at the cost of deception. In this context, social media communication reduces the verifiability of the accuracy of photographs with the consequence that the accuracy of the photograph is hard to verify. This contradicts the general trend [1], that social media enhance the likelihood of genuine communication and that the truth of messages is relatively easy to verify.

Photo editing behavior increases the options available for self-presentation on social network sites and constitutes a significant restriction on verifiability of the accuracy of profile photos. In addition, it is likely to be negatively correlated with self-perceived attractiveness. Therefore, the importance of photo editing behavior in the context of social network sites is high.

Photo editing behavior represents an emerging trend. According to a survey of the Renfrew Center Foundation [4], 50% of SNS users edit their photos before posting them to Social Network Sites (SNSs). Still, the effects of these new photo editing applications on the individual are largely unknown.

Compared to past decades, people are nowadays constantly confronted with highly edited beauty pictures on SNSs, which could significantly change the perception of beauty by raising beauty standards. Accordingly, individuals of average attractiveness may perceive themselves as less attractive when evaluated in comparison with photos of more attractive individuals which were edited by photo editing behavior. Analogous contrast effects have been found in a field study in which a moderately attractive woman was evaluated less positively following exposure to highly attractive actresses [5, 6]. In addition, social comparisons on social media are likely to impair self-esteem [7].

What happens when the comparison is made with a more beautiful and optimized version of oneself? Numerous studies indicate a negative association between photo editing behavior on SNSs and body satisfaction [8-13]. Furthermore, users who retouched their pictures reported feeling less attractive, poorer self-esteem [12], and increased negative mood ([13]. Photo editing behavior may also encourage individuals to view their body as an object [14] reinforcing associated risks such as body shame, depression, and eating disorder [15].

The aim of this research is to reveal risks of the engagement in photo editing behavior. For this purpose, various factors identified in previous studies were incorporated into a parallel-sequential mediation model with multiple mediators. From a theoretical point of view, we integrate selfie editing, social comparisons, self-objectification, and well-being which is captured by self-esteem.

Whereas almost all previous studies have investigated the impact of photo editing behavior solely on body image, this study refers to self-perceived attractiveness in general by including the body *and* face as part of self-perceived attractiveness. One reason is that, so far, filters focus on the face and not on the body. Also, users post more pictures of their face on SNSs than full body pictures [16]. The face is usually more salient in pictures of oneself [13]. Thus, photo editing may lead individuals to pay closer attention to their facial attractiveness. Accordingly, photo editing behavior can have a significant effect on facial dissatisfaction, but not on body dissatisfaction [13].

Additionally, both men and women are included in this study. To date, almost all studies on photo editing have only included women, as they are more likely to engage in photo editing behavior [17] and experience higher pressure to conform to the cultural beauty ideal [18]. Yet, [10] found that photo editing behavior was positively associated with body dissatisfaction for both genders. The effects of photo editing behavior for men are nevertheless nearly unexplored.

## Theoretical background

### Photo editing behavior

Photo editing behavior refers to the use of filters as well as various photo editing applications. While filtering options within Instagram change the face using a template with features, such as makeup, enlarged eyes, fuller lips, and narrower noses, photo editing applications provide more specific options. Thus, users can specifically select which parts of their face and body they want to edit. The functions range from changing skin tones, removing blemishes, slimming faces, making body parts slimmer, making body parts appear bigger, changing the shapes of noses, lips, cheeks, chins and eyes, and various makeup options.

Moreover, there are photo editing applications that use artificial intelligence (AI) to fully reconfigure the face [19]. While the use of photo editing options is mostly self-determined, as the user consciously decides which physical features should be changed, the use of filters or AI provides less self-determination. In this case, it is not the user but the technology that determines which of the photo’s physical features require modification. This could cause users to discover flaws in themselves that they would not have noticed without using the photo editing application.

As the physical appearance of users plays an important role in impression management on SNSs, photo editing behavior serves as an impression management strategy of online self-presentation [20] besides, for example, selecting one’s best photo. Regarding self-presentation, users can manage the impressions they have on others by minimizing perceived flaws or imperfections to get more favorable attention from others [3]. However, the use of photo editing applications can create an unrealistic expectation of one’s own attractiveness [21].

### Self-perceived attractiveness

Self-perceived attractiveness refers to people’s beliefs about the quality of their physical appearance [22]. In contrast to body image, self-perceived attractiveness involves not only the perception of one’s own body but also of one’s face.

Several studies revealed a positive correlation between photo editing behavior and body dissatisfaction [8-13], whereas others found no significant association [8, 11]. Overall, research has suggested that photo editing behavior may represent a risky behavior in terms of its potential to negatively impact body image [12] and facial satisfaction [13]. In addition, higher involvement in photo editing behavior, but not higher media exposure, is associated with higher body dissatisfaction [11]. Therefore, the importance of the general level of media exposure as a potential confounding variable is likely to by small. A plausible explanation of these results is that photo editing makes users think more about their flaws and imperfections [12]. Thus, individuals who engage in photo editing behavior are unfortunately more likely to notice a gap between their actual and ideal appearance [23]. This is likely to diminish self-perceived attractiveness in terms of appearance. Conversely, it can be argued that low self-perceived attractiveness tends to elicit photo editing behavior [24]. Accordingly, the first hypothesis states:

#### H1

Photo editing behavior is negatively correlated with self-perceived attractiveness in terms of appearance.

### Self-objectification

As individuals engaging in photo editing behavior focus more on their appearance [16], it is tempting for them to anticipate the reactions of other users to the edited photo and look at themselves from an outside viewers’ perspective. Since the focus on many SNSs is on the user’s appearance, SNS users tend to expect to be evaluated based on their appearance [25]. Both conditions are risk factors for self-objectification.

Self-objectification is defined as the act of “[internalizing] an observer’s perspective on self” ([15] pp. 179 f.). The difference between self-objectification and body dissatisfaction is that self-objectification is a perspective toward the body, whereas body dissatisfaction involves negative feelings about one’s body [26]. As the objectification theory originally included only women, it was argued that women frequently experience sexual objectification by being valued for their appearance or by being regarded as objects. This regular experience of sexual objectification, such as exposure to objectifying media, socializes women to internalize an outside viewers’ perspective on their appearance [27]. Consequently, when a woman self-objectifies, she thinks about how her body might look to others [15]. The negative consequences of such an approach may include, among others, body dissatisfaction, body shame, disordered eating [28], depression [26], and lower well-being [29]. Meanwhile, it is proven that also men experience self-objectification and are therefore equally exposed to these risks [27].

In general, the nature of photo editing behavior activates feelings of self-objectification [12; 14] and physical appearance comparisons. Taking an outsider’s perspective makes users focus on their appearance rather than unobservable attributes such as abilities [16, 27, 30]. Additionally, photo editing behavior reinforces the evaluation of their appearance [31]. [32] argued that self-objectification can be triggered when people spend time editing their own photos because they view themselves in photos as manipulated objects. Furthermore, [33] proposed the circle of objectification, which suggests that individuals who self-objectify seek out more appearance comparisons, which in turn acerbate tendencies of self-objectification, as appearance comparisons increase the salience of one’s appearance [34]. Therefore, the positive association between photo editing behavior and self-objectivation may also be triggered by physical appearance comparisons resulting from self-objectification (cf., Sect. 2.4). Based on former studies, we derived as replication hypothesis:

#### H2a

Photo editing behavior is positively correlated with self-objectification.

### Physical appearance comparisons

In general, self-objectification is closely linked to appearance comparisons because both constructs share the perspective toward the body. According to social comparison theory, humans have an innate drive to compare themselves with others as a source for self-evaluation [35]. This happens relatively automatically. While social comparisons include abilities, affect, self-esteem, performance satisfaction, and other personal characteristics [36], physical appearance comparisons focus on physical characteristics [37]. In upward comparisons, the individual evaluates her- or himself relative to someone who is considered more attractive. [36].

During photo editing, users compare their own appearance to sociocultural beauty standards and might think about the required modification through photo editing to get closer to this ideal [38]. Therefore, photo editing behavior is likely to be positively associated with physical appearance comparisons.

#### H2b

Photo editing behavior is positively correlated with physical appearance comparisons.

In general, social comparisons tend to elicit contrast effects [36]. Therefore, upward comparisons are likely to reduce appearance evaluation. Individuals of average attractiveness may be perceived as less attractive when evaluated in comparison with more attractive individuals. Therefore, one’s tendency to engage in physical appearance comparisons is likely to be associated with body dissatisfaction [39], internalization of appearance ideals, low self-esteem, sexual objectification, body surveillance, and body shame [37]. Similar contrast effects have been demonstrated when a moderately attractive individual is evaluated following exposure to highly attractive media stimuli [40]. Moreover, individuals who perceive themselves as less attractive are more likely to engage in physical appearance comparisons and upward comparisons (Patrick et al., 2004). Although in theory individuals who are satisfied with their body may engage frequently in physical appearance comparisons, the empirical evidence reveals that individuals who perceive themselves as less attractive primarily engage in physical appearance comparisons, more than individuals who perceive themselves as attractive [41]. Therefore, empirical results and theoretical considerations lead to the following hypothesis:

#### H3

Physical appearance comparisons are negatively correlated with self-perceived attractiveness in terms of appearance.

According to the self-discrepancy theory, individuals compare one self-state to another self-state and find that a discrepancy exists [42]. This discrepancy in turn triggers dissatisfaction. Therefore, the negative effects of photo editing behavior are likely to occur when SNS users perceive high discrepancy between their edited self (i.e., the idealized self) and real self [13; 42]. It is quite likely that the individual will fall short of the unrealistic beauty ideal promoted by filters and photo editing applications, resulting in a body-related self-discrepancy [43].

### Self-esteem

Individuals who express low self-esteem more often gravitate to physical appearance comparisons, seeking reassurance and validation compared with individuals high on self-esteem. However, they also more often engage in upward comparisons [44], which in turn is associated to a decrease in self-esteem [37]. Such upward comparisons may serve as a reminder of the beauty ideal they do not meet [41] eliciting a contrast effect.

Self-esteem represents an important part of subjective well-being. It is defined as the affective-evaluative facet of the self and includes cognitive-knowledge-based, affective-evaluative, and action guiding facets [45]. Moreover, self-perceived attractiveness is an important component of self-esteem. Numerous studies indicated a positive correlation between self-perceived attractiveness and self-esteem [44, 46-49], as it is an important source of power and social status [50] Also, attractive individuals develop and internalize more positive self-views than less attractive people [51]. Several researchers have argued that it is not attractiveness itself that is associated with self-esteem, but individuals’ evaluation of their own attractiveness [52]. In conclusion, self-perceived attractiveness is likely to play an important role in determining self-esteem, possibly more important than objective attractiveness. This conclusion does not imply that individuals have only a vague idea of their objective attractiveness or no idea at all. There is definitely objectivity regarding self-perceived facial attractiveness [53]. Therefore, objective facial features affect self-perceived facial attractiveness. The fact that objective facial attractiveness is registered by individuals suggests that objective facial attractiveness may constitute a confounding factor with respect to self- esteem that could influence the results of the study (cf., the Discussion section). Nevertheless, self-perceived attractiveness is associated with self-esteem beyond objective facial attractiveness having a positive impact on self-confidence [54]. Moreover, individuals with high self-esteem are more likely to accept their physical appearance [52]. This reasoning leads to the following hypotheses:

#### H4

Self-perceived attractiveness in terms of appearance is positively correlated with self-esteem.

In sum, photo editing behavior leads to self-objectification [12; 14; 20; 32] and physical appearance comparisons [24]. A possible explanation is that photo editing behavior makes users focus more on their appearance, which consequently increases physical appearance comparisons [16]. In turn, physical appearance comparisons are likely to trigger photo editing behavior to compensate for one’s optical flaws (cf., the occurrence of objective facial attractiveness; [53]). Since physical appearance comparisons [39, 55] and self-objectification [28] are negatively correlated with body image and self-perceived attractiveness, it is reasonable to assume that the two constructs are mediators of the relationship between photo editing behavior and self-perceived attractiveness. When individuals self-objectify and compare themselves regarding their physical appearance, they may pay more attention to their physical appearance and more easily find a gap between their physical appearance and their beauty ideal. As a result, they presumably feel dissatisfied with their own appearance [30].

Finally, in *H5* a parallel-sequential multiple mediation model is proposed, which is based on previous results in general and *H1* to *H4* in particular. The model connects photo editing behavior with self-esteem via three mediators.

#### H5

Photo editing behavior is associated with higher self-objectification and more physical appearance comparisons, that result in lower self-perceived attractiveness, which, in turn, implies lower self-esteem.

**Fig. 1:**
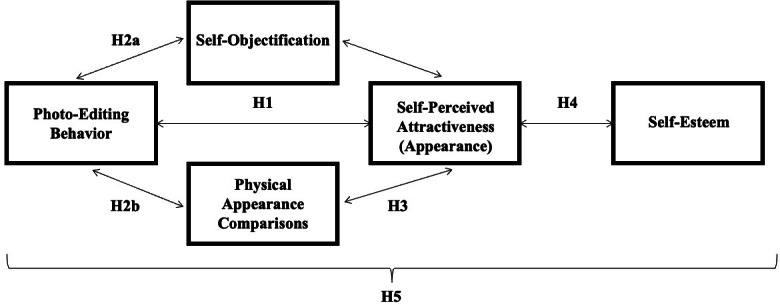
Overview of the research plan including hypotheses

An overview of the research plan including the hypotheses^1^ is shown in Fig. 1.

## Method

### Sample

The program G*Power (version 3.1.9.7; [56]) was used to calculate in advance how many participants constitute a sufficient sample size for a mediation model (i.e., multiple regression with 5 predictors). The significance level was set at 5%, Power (1-ß) at 95%. Furthermore, small to medium effect sizes were assumed, f^2^ = 0.15 since in social psychological research these effect sizes are to be expected [57]. The appropriate sample size turned out to be *N* = 138. 403 participants met the inclusion criteria and provided complete data sets. 316 (78.4%) were female, 85 (21.1%) male and 2 (0.5%) identified as neither female nor male. The average age was 27.6 years (*SD* = 8.3) ranging from 18 up to 61 years. 42.2% held an academic degree and 53.3% were at least high school graduates. 86.6% of the participants were students and most of them studied psychology (59.1%).

### Procedure

An online survey was conducted via Unipark (https://www.unipark.de). Participation was dependent on being active on SNSs in general. More specifically, the sample is based on Instagram members because Instagram is the most widely used SNS [44], primarily image-based compared to other SNSs [13], and has its own photo editing features. To confirm the inclusion criteria, participants were asked if they had an Instagram account. Therefore, the likelihood of engaging in photo editing behavior was expected to be relatively high for Instagram users. Furthermore, participants had to be at least 18 years old. They were recruited via social media posts following a snowball-sampling technique.

### Questionnaires

Demographic variables, photo editing behavior, self-objectification, physical appearance comparison, self-perceived attractiveness and self-esteem, and Instagram activity were obtained. In general, higher scores indicate higher levels of the corresponding variable.

#### Photo editing scale

The Photo Editing Scale (PES) was newly developed to measure photo editing behavior related to the use of SNSs. It provides a brief measure and consists of five items, each of which are answered on a 5-point Likert scale with a response format ranging from 1 = “never” to 5 = “always”. To assess different types of photo editing behavior, participants were asked about their use of filters (e.g., “I use filters that beautify my facial features.”) and photo editing applications (e.g., “I edit my facial features before uploading a photo (using apps such as Facetune or Faceapp, for example”). One item was included focusing on body features (e.g., “I edit my figure before I upload a photo (e.g., with apps like Facetune or Faceapp)”). Two further items referred in general to using photo editing or not. Reliability analysis indicated a satisfactory internal consistency, α = 0.75, given the small number of items. The response scale of one item had to be inverted. Higher scores represent more photo editing behavior. On average, participants exhibited relatively low photo editing behavior-scores, *M* = 1.89, *SD* = 0.79. Note that the content validity of the items is high. The scale items are included in OSF (with respect to the double-blinded review process, the link will be added after acceptance of the paper).

#### Instagram activity questionnaire

To measure Instagram activity, the Instagram Activity Questionnaire (IAQ; [58]) was used. The questionnaire consists of 38 items rated on a 5-point Likert scale from 1 = “never” to 5 = ”very often”, based on the two factors: *Active* (27 items; e.g., “I post pictures.”) and *Passive* (11 items; e.g., “I look at the photos of other users.”). Reliability analyses indicated very good internal consistencies (α_IAQ_ = 0.91, α_Active_ = 0.88, α_Passive_ = 0.82). Higher scores indicate higher Instagram activity. Participants exhibited moderately high ratings of Instagram activity, i.e., *M*_IAQTotal_ = 2.64, *SD*_IAQ Total_ = 0.57, *M*_Active_ = 2.52, *SD*_Active_ = 0.64, *M*_Passive_ = 2.91, *SD*_Passive_ = 0.67, with highest ratings for passive Instagram use.

#### Self-objectification beliefs and behaviors scale

For measuring self-objectification, the Self-Objectification Beliefs and Behaviors Scale (SOBBS; [26]) was used. This is a relatively new measure that addresses the primary limitations of existing measures. Participants were asked to rate their level of agreement with each item using a 5-point Likert scale from 1 = “strongly disagree” to 5 = “strongly agree”. The 14 items are based on two factors: (1) *internalizing an observer’s perspective of the body* (7 items; e.g., “I try to imagine what my body looks like to others (i.e., like I am looking at myself from the outside)” and (2) *equating the body to who one is as a person and valuing physical appearance above other attributes* (7 items; e.g., “How I look is more important to me than how I think or feel.”). For the present German sample, the SOBBS was translated and validated by using the back-translation procedure (see Appendix A). The measure demonstrated very good internal consistencies in the original study [26] as well as in the current sample, α_SOBBS_Total_ = 0.89, α_Factor_1_ = 0.89, α_Factor_2_ = 0.84. Higher scores indicate more self-objectification. In general, participants exhibited moderately high ratings of self-objectification, *M*_Factor_1_ = 2.92, *SD*_Factor_1_ = 0.88, *M*_Factor_2_ = 1.70, *SD*_Factor_2_ = 0.62, *M*_SOBBS_Total_ = 2.31, *SD*_SOBBS_Total_ = 0.66, with highest ratings on the first factor (internalizing an observer’s perspective of the body) and comparatively low ratings on the second factor (equating the body to who one is as a person and valuing physical appearance above other attributes).

#### Physical appearance comparison scale

The German version [59] of the Physical Appearance Comparison Scale (PACS; [60]) was used to assess an overall tendency to compare one’s own appearance with others (e.g., ‘‘In social situations, I sometimes compare my figure to the figures of other people’’). With only 5 items, the PACS is a very economical measure. Items are rated on a 5-point Likert scale (1 = “never” to 4 = “always”). Higher mean scores indicate higher frequency of physical appearance comparisons. As the most widely used measure for physical appearance comparisons, the PACS has demonstrated good reliability and validity (Schaefer & Thompson, 2014). Accordingly, [59] have found that the internal consistency of the scale based on a German sample is within an acceptable range. The current investigation revealed moderately high levels of physical appearance comparisons, *M* = 2.73, *SD* = 0.79 and a satisfactory internal consistency, α = 0.73.

#### Body-esteem scale

Self-perceived attractiveness was measured with the Body-Esteem Scale (BES) [61] that consists of 23 items rated on a 5-point Likert scale ranging from 1 = “never” to 5 = “always”. [61] suggested that feelings about one’s weight can be differentiated from feelings about one’s general appearance. Also, one’s own opinions may be differentiated from the opinions attributed to others. Therefore, the BES contains three subscales: (a) *Appearance* (6 items; e.g., “I wish I looked better”), (b) *Weight* (4 items; e.g., “I really like what I weigh”) and (c) *Attribution* (4 items; e.g., “People of my own age like my looks”). These subscales measure (a) general feelings about one’s own appearance, (b) weight satisfaction, and (c) evaluations attributed to others about one’s appearance [46]. For the present research, only the subscale on *Appearance* was used. Hence, 6 of the 14 items of the questionnaire were included and translated into German. The translation was verified through back-translation procedure (see Appendix B). There is good evidence that the original scale is valid and reliable over a wide age range [61]. Reliability analyses in the current sample indicated that internal consistency was excellent, α_Appearance_ = 0.91. On average, participants ratings regarding their self-perceived attractiveness were moderately high, *M*_Appearance_ = 2.72, *SD*_Appearance_ = 0.74.

#### Rosenberg self-esteem scale

For measuring self-esteem, the German version of the Rosenberg Self-Esteem Scale (RSES) [62] was used, which showed good reliability and validity [63]. With respect to construct validity, strong positive correlations between self-esteem and both self-satisfaction and self-efficacy and a substantial negative correlation between self-esteem and self-derogation were obtained. The RSES includes six items rated on a 4-point Likert scale from 1 = “strongly disagree” to 4 = “strongly agree*”*. Positive and negative feelings about the self were both assessed. The response scales of five of the ten items had to be inverted (e.g., “Every now and then I think I’m no good at all.“). Higher scores indicate higher self-esteem. The RSES is characterized by easy and quick use and high face validity [64]. [65] reported a satisfactory internal consistency for the German version of the RSES. In the current sample, reliability analyses indicated a very good internal consistency, α = 0.90. On average, participants reported relatively high self-esteem, *M* = 3.21, *SD* = 0.61.

## Results

### Preliminary analysis

As indicated by Kolmogorov–Smirnov test and Q–Q plots, some of the scales (PES, BES, RSES) were not normally distributed. Consequently, Spearman’s rank correlation (Rho), which presupposes an ordinal association between variables, was used for all computations of their interrelations.

#### Validity checks

In general, the content validity of the employed scales is high. The construct validity of each of the scales included in hypothesis tests was scrutinized thoroughly except for the self-esteem scale whose construct validity was comprehensively demonstrated by [63]. For checking construct validity of the PES, it was correlated with the IAQ because Instagram use is likely to promote appearance concerns [12] and in this respect corresponds with the PES because of its visual, photo-oriented features. Therefore, a positive correlation between the IAQ and PES was expected. This expectation was confirmed because the IAQ was positively correlated with the PES, *r*_*s*_ (401) = 0.358, *p* < .001, and its subscales, both *Active, r*_*s*_ (401) = 0.207, *p* < .001, and *Passive, r*_*s*_ (401) = 0.223, *p* < .001. Note that the highest correlation was displayed between PES and the total Instagram Activity Questionnaire accounting for 12.8% of common variance.

Subsequently, the construct validity of the German version of the Appearance subscale of the Body Esteem Scale was examined by correlating it with the passive subscale of the Instagram Activity Scale. High exposure to Instagram content in a passive mode is likely to undermine positive attitudes toward own appearance because of the elicitation of social comparisons which lead to body dissatisfaction [18]. Results correspond with the assumption of construct validity of the Appearance subscale, *r*_*s*_ (401) = − 0.135 *p* < .01, indicating that higher passive Instagram consumption is negatively associated with positive attitudes toward own appearance.

Additionally, the construct validity of the Self-objectification Beliefs and Behaviors Scale (SOBBS) was examined by correlating it with the Body Esteem Scale (BES) because higher self-objectification seems to imply lower self-perceived attractiveness [66]. In correspondence with expectations significant negative correlations were found between self-perceived attractiveness and self-objectification, *r*_*s*_ (401) = − 0.569 *p* < .001, as well as on corresponding subscales, referring to *internalizing an observer’s perspective of the body*, *r*_*s*_ (401) = − 0.462, *p* < .001, and referring to *equating the body to who one is as a person and valuing physical appearance above other attributes*), *r*_*s*_ (401) = − 0.477, *p* < .001), respectively. The highest correlation was exhibited between the total SOBBS and perceived attractiveness. indicating 23.9% of common variance.

Finally, the construct validity of the Physical Appearance Comparison Scale was examined. In accordance with results by [7, 67] indicating that social comparison orientation is positively associated with Facebook activity it was assumed that the PACS is positively linked with Instagram activity substituting comparison orientation by the Physical Appearance Comparison Scale and Facebook activity by Instagram activity. Note that comparison orientation and PACS both represent individual-difference measures of the readiness to perform social comparisons and that the Instagram activity questionnaire was developed analogously with the Facebook activity questionnaire. Both instruments capture behavioral reports of activities on SNSs. In correspondence with expectations and corroborating the construct validity of the PACS results indicated that total Instagram activity and PACS are correlated positively, *r*_*s*_ (401) = 0.264, *p* < .01. In addition, the results for both active and passive Instagram activity measures correspond with the results for the overall activity measure.

### Testing hypotheses

The hypotheses pertain to correlational relationships. The complete intercorrelation matrix is summarized in Appendix C. In *H1* it was hypothesized that photo editing behavior is negatively correlated with self-perceived attractiveness. This hypothesis was confirmed because a significant negative correlation was found between photo editing behavior and self-perceived attractiveness in terms of appearance, *r*_*s*_ (401) = − 0.146, *p* < .01.

As hypothesized in *H2a*, photo editing behavior displayed a significantly positive correlation with the SOBBS measuring self-objectification, *r*_*s*_ (401) = 0.221, *p* < .001. This correlation represents a moderate effect. Moreover, with respect to the SOBBS-subscales, substantial negative correlations were found between photo editing behavior and *internalizing an observer’s perspective of the body*, *r*_*s*_ (401) = 0.227, *p* < .001, and *equating the body to who one is as a person and valuing physical appearance above other attributes*, *r*_*s*_ (401) = 0.124, *p* < .001. Therefore, *H2* was confirmed.

Furthermore, the proposition (*H2b*) was investigated that photo editing behavior is positively associated with physical appearance comparisons. The results corresponded with *H3*, *r*_*s*_ (401) = 0.238, *p* < .01. More specifically, participants who were more active in terms of photo editing behavior exhibited more physical appearance comparisons.

*H3* postulated a negative association between physical appearance comparisons and self-perceived attractiveness in terms of appearance. It was corroborated by a significant negative correlation between the subscale *Appearance* of the BES and physical appearance comparisons, *r*_*s*_ (401) = − 0.536, *p* < .001, Therefore, *H4* was confirmed.

*H4* refers to the association between self-perceived attractiveness and self-esteem. Previous research demonstrated that self-perceived attractiveness and self-esteem are positively linked [44; 49]. Supporting previous results, self-perceived attractiveness and self-esteem were correlated positively. Specifically, the BES-subscale *Appearance* was positively associated with self-esteem, *r*_*s*_ (401) = 0.604, *p* < .001, representing a strong effect. Thus, *H5* was confirmed by the results.

**Fig. 2:**
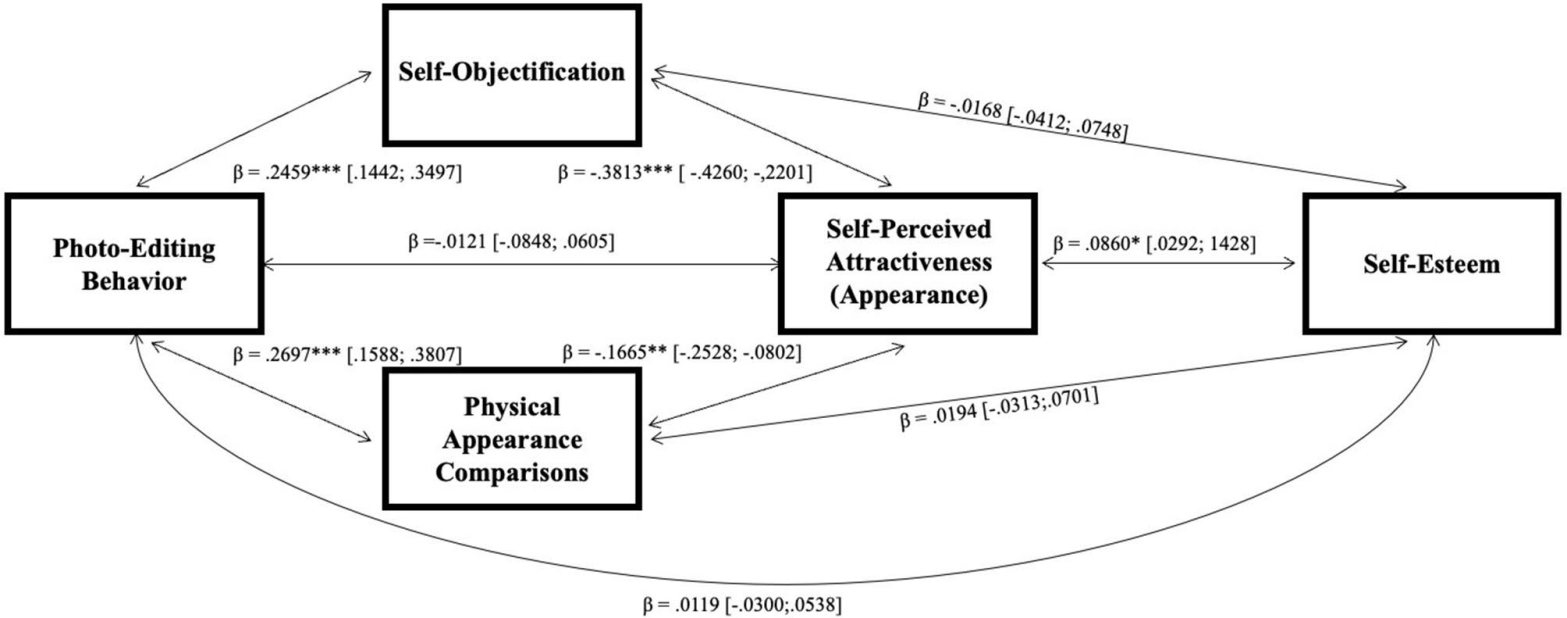
Mediation model. Note: m = 10,000; bootstrapping intervals in brackets; Age (ß = 0.015, *p* > .05; CI [-0.0445; 0.0679]) and gender (ß = 0.029, *p* > .05; CI [-0.0015; 0.0036]) as covariates show no significant effect on the statistical mediation model, all *p*s > 0.05. *p < .05, **p < .01. ***p < .001

The statistical mediation model summarized in *H5* proposed that photo editing behavior is associated with higher self-objectification and more physical appearance comparisons, and that both mediators are associated with a lower self-perceived attractiveness, which, in turn, is associated with lower self-esteem. Note that the mediators self-objectification and physical appearance comparisons display a high positive correlation which is taken into account by applying a path-analytic model. In overview, the corresponding path analysis summarized in Fig. 2, revealed a significant parallel-sequential multiple mediation in the expected direction, total indirect effect: *β* = − 0.020, BC 95% CI [-0.0377; − 0.0063]; overall model: *F*_4,398_ = 7.12, *p* < .001, adj *R*^*2*^ = 0.067. While the direct effect of photo editing behavior on self-esteem was not significant, *β* = 0.012, BC 95% CI [-0.0300; 0.0538], the indirect effect was mediated via self-objectification, physical appearance comparisons, and self-perceived attractiveness. Therefore, *H5* was confirmed. Photo editing behavior significantly predicted more self-objectification as well as more physical appearance comparisons which both predicted lower self-perceived attractiveness and lower self-esteem. In addition, self-objectification directly predicted lower self-esteem.

### Post-hoc tests of observed power and replicability

We used the *test of excessive significance* (TES) [68] to calculate the success rate, median observed power, the inflation rate, the replicability index, and a test of insufficient variance (TIVA) based on 6 hypotheses-oriented effects (i.e., 5 *t-*statistics and 1 *F*-statistics based on intergroup deviations and mediation models). Therefore, we used the *p-checker-app* (see http://shinyapps.org/apps/p-checker/*).*

The TES revealed a success rate of 1, which indicates that 100% of our predictions were confirmed, and a median observed power of 99.6. In addition, the TES revealed a minimal inflation rate 0.004, which indicates that no more hypotheses have been confirmed than possible under consideration of the power. The *r-index* = 0.99 indicates that our findings can be (theoretically) replicated in X * 0.99 follow-up studies.

At last, the TIVA, X^2^ (5) = 98.311, *p* = 1, var = 19.66, indicated that no bias was present confirming that all entered test statistics and p-values are in the expected direction.

## Discussion

The hypotheses connected the construct of photo editing with social comparison, self-objectification, and self-esteem as an indicator of well-being. Photo editing includes selfie editing as a special case. Whereas *H1* to *H4* postulated associations between two constructs, *H5* combined five constructs based on a path-analytic model. In general, our findings support the hypotheses.

Consistent with other studies [8-13], the results regarding *H1* indicate that photo editing behavior is associated with lower self-perceived attractiveness in terms of appearance. Although the explained variance is rather small, the corresponding correlation is highly significant. One explanation is that individuals who often edit their pictures create an idealized virtual self-image which enhances the discrepancy between the real and ideal self [24]. Furthermore, even people satisfied with their appearance presumably want to look even better and edit their selfies to post perfect ones which maximize ideal online self-presentation [24, 53].

As expected in *H2a*, a significant positive correlation between photo editing behavior and self-objectification was found. On the one hand, self-objectification may predispose individuals to engage in photo editing behavior. On the other hand, photo editing behavior is likely to enhance feelings of self-objectification [12, 14], as the individual simultaneously becomes the editor and the object of photo editing in general and selfie editing in particular. Self-objectification may foster an individual’s need to constantly present and improve his or her physical appearance to please others [14]. Therefore, people with a higher degree of self-objectification may place a higher value on posting photos that reflect the societal beauty ideal. Individuals who self-objectify are more likely to experience body shame and body dissatisfaction [28], Editing specific body parts may reduce the body to its component parts rather than viewing it as a fully functioning whole. As filters and photo editing applications tend to convey beauty ideals, the internalization of these messages may guide the perception of one’s appearance, leading to a more objectified view. [69] reported that time spent on SNSs was associated with higher self-objectification. Therefore, the correlation between photo editing behavior and self-objectification may be intensified by higher length of SNS use.

*H2b* postulated a positive association between photo editing behavior and physical appearance comparisons. The confirmation of *H2b* indicates that higher sores on the photo editing scale are associated with more intense immersion in physical appearance comparisons including the construction of self-other contrasts with respect to good looks. Note that the confirmation of both *H2a* and *H2b* in combination emphasizes that self-objectification and physical appearance comparisons are closely linked with each other. The correlation between both scales is substantial, *r*(401) = − 0.599, *p* > .001. Therefore, the confirmation of *H2* taken together supports the notion that photo editing behavior is associated with change in the perspective toward the body.

The findings generally supported hypothesis *H3* that physical appearance comparisons are negatively associated with self-perceived attractiveness in terms of the subscale *Appearance* of the BES. To explain these findings, it should be noted that such comparisons may serve as a reminder of beauty ideals that one does not meet [41]. This correlation should be particularly pronounced for SNS users, as upward physical appearance comparisons are likely to occur frequently on SNSs due to a general tendency of users to exaggerate their positive characteristics striving for positive self-presentation [70]. Intriguingly, physical appearance comparisons with peers may actually impair self-perceived attractiveness more than comparisons with fashion models, because the latter are perceived as less similar to oneself and, as a consequence, represent a less diagnostic comparison group [71]. Due to the high similarity of an optimized version of oneself to one’s real self, physical appearance comparisons with one’s artificially optimized self could have a negative effect on self-perceived attractiveness. These comparisons reveal what needs to be optimized to achieve the ideal. For example, lip injections, nose surgery, anti-wrinkle cream or weight loss could presumably make the edited selfie self-achievable, while in comparison, the appearance of a celebrity appears to be unachievable. People don’t recognize that the appearance of celebrities is usually artificially enhanced using make-up and software like *Adobe Photoshop*.

Individual differences in responses to physical appearance comparisons are likely. Specifically, upward comparisons could inspire some individuals, whereas others may feel discouraged. In accordance, [41] argued that reactions to physical appearance comparisons are largely a function of two individual differences: The extent to which one’s self-esteem is contingency based and one’s self-perceived attractiveness.

Several studies have already shown a positive association between self-perceived attractiveness and self-esteem [44, 46-49]. The confirmation of *H4* which states that self-perceived attractiveness is positively associated with self-esteem is in line with previous results. Highly attractive individuals are likely to internalize more positive self-views than less attractive people [51]. Interindividual differences should also be considered, as some people are more likely to define their self-esteem on the basis of meeting expectations such as societal beauty ideals [72]. This refers to the contingent self-esteem, which is based on the approval of others or on social comparisons [73]. Individuals who are more dependent on contingent self-esteem may be more concerned with attractiveness than others who, for example, rely more on academic success or social acceptance [41].

Self-esteem is likely to be influenced by both self-perceived attractiveness and objective attractiveness [53]. Therefore, objective attractiveness may constitute a confounding factor with respect to the link between self-perceived attractiveness and self-esteem because higher objective attractiveness could be associated both with both self-perceived attractiveness and self-esteem. Future studies, which should include a measure of objective attractiveness, could clarify this issue. Nevertheless, stereotype research indicates [74] that cultural reference systems and subjective impressions represent powerful determinants of self-esteem.

In general, the confirmation of *H1 to H4* represents initial evidence for the mediation model postulated in *H5*. But *H5* goes beyond the other hypotheses by specifying specific paths which connect photo editing behavior with self-esteem. The employment of the sequential multiple mediator model in testing *H5* allows to discover these paths. The results indicate that mediation via self-objectification and via physical appearance comparisons occupy central switching points in the model which are both associated with self-perceived attractiveness. Therefore, the link between photo editing behavior and self-esteem was sequentially mediated via self-objectification, physical appearance comparisons, and self-perceived attractiveness. Individuals who engage in photo editing behavior more often perform physical attractiveness comparisons with others sand self-objectify more frequently. Whereas self-objectification relates to self-esteem both indirectly via self-perceived attractiveness and directly, physical appearance comparisons are only indirectly connected with self-esteem via self-perceived attractiveness.

The path-model specifies links from photo editing behavior to restricted self-esteem by focusing on unintended side-effects of photo editing behavior which is performed mainly to achieve positive consequences (e.g., improved self-presentation). From an applied viewpoint it would be desirable to inform users about the danger that such side-effects may occur. Such a cautionary note might include a broader concern related to the improvement of appearance in public. For example, people don’t recognize that the appearance of celebrities is usually artificially enhanced using make-up and software like *Adobe Photoshop*. Therefore, photo editing of selfies on SNSs is only one instance of a general trend to edit pictures. Reality is more elusive as it appears on the surface. The depiction of reality is a constructive endeavor which is subject to concealed issues of the editors. The depiction of reality is usually not a documentary but part of a narrative which the photo editor intends to project on the public screen. By understanding the underlying narrative, the contrast between natural appearance and edited photo of it is getting transparent. Because photo editing is likely to prevail in the future, the focus of psychoeducation as part of a psychological intervention technique should be a sensibilization for the wide spread of use of corresponding techniques.

## Limitations and future research

This study is subject to several limitations. Firstly, the sample is not representative. For example, the data was obtained within an online context. But research indicates that differences in results occur between offline and online contexts. Specifically, the occurrence of gender differences in personality depended on the context of measurement [83, 84]. Therefore, in future studies the results of online and offline measurement of the assessment of photo editing variables including personality variables like self-esteem should be compared with each other in order to increase the generalizabilitv of results.

In addition, young German participants were overrepresented. But the sample comprises individuals within a large age range and from different socioeconomic and academic backgrounds. Note that age plays a major role in the perception of facial attractiveness and self-esteem[75]. The present sample includes a range of individuals between 18 and 61 years old, with 86.65% being students, and thus more likely in their twenties. To account for doubts with respect to its representativity regarding the general population we added subanalyses including age as covariate in our mediation model. However, the results confirmed our previous results.

Furthermore, 59% of the participants were psychology students and only 21% of the participants were male. the high proportion of females in the sample could mean that the results are more typical for females than for males. In fact, it was found that women are more involved in photo editing behavior than men [16; 17], are more preoccupied with appearance than men [76], and experience higher pressure to conform to the societal beauty ideal [18]. Note that we added gender as covariate in our mediation model. No significant effects were found.

Another issue that goes beyond sample characteristics is that most filters focus primarily on realizing the female beauty ideal.

The variables measured in this study are based on self-report. Therefore, they may be influences by response biases. For example, it is important to note that participants may have underreported their photo editing behavior because they may have perceived this behavior as socially undesirable. In support of this argument, previous research found that 12% of photos posted under the #nofilter tag on Instagram did in fact include filters [77]. Therefore, future research could benefit from inclusion of a measure of social desirability. In defense of the data quality of the self-report scales, we investigated the construct validity of the scales. Results indicated that each of the variables which were represented in the hypotheses exhibit substantial construct validity. In addition, the content validity of all scales is high.

Additionally, the Body Esteem [61] is validated for individuals between 12 and 25 years of age. We suggest that the scale is validated in older populations. This is in line with our results showing the same effects with respect to our hypotheses regardless of adding age as covariate.

Please note that participation was based on having an Instagram account. There are reports in the literature regarding the percentage of individuals who edit their photos before publishing them on Instagram. These range between 30 and 90%. It can by assumed that the sample also included individuals who have no experience in photo editing, although they have an Instagram account. This was also evident by the low photo editing behavior score of the participants in this study. Nonetheless, although we determined a low score on photo editing behavior in our study, we found robust results confirming our hypotheses.

Based on sample characteristics (i.e., age, gender, and participants’s photo editing behavior), some points of criticism with respect to the generalizability of our data arise. However, according to our mediation analyses including age and gender as covariates these variables had no significant confounding effect on our results. Additionally, we calculated further post-hoc analyses with respect to replicability, post-hoc power as well as insufficient variance showing that our data seem to be replicable, unbiased, and generalizable. However, future studies with a more balanced sample are necessary to confirm our findings.

In addition, the statistical analyses in this study are correlational, meaning that no causal conclusions are warranted. Given the early phase of research on photo editing, this restriction may be acceptable. Furthermore, significant mediation does not imply true mediation but only that the data fits with the proposed mediation model [78]. Future studies are needed to examine causal inferences. For example, is photo editing behavior the cause of more self-objectification or vice versa? Such questions might be tackled by experimental studies [79], with respect to the negative impact of social comparisons on self-esteem) or longitudinal research design [80], with respect to effects of social media use on mental health).

Finally, the list of potential mediators between photo editing behavior and self-esteem includes variables that were not considered in our research design. It may include appearance contingent self-esteem [81], upward and downward social comparisons [24], and narcissism [82] Time spent on SNSs should be included as a possible confounding variable in future research. Furthermore, research should be conducted to determine the extent to which reactions to edited photos in the form of likes, comments, or compliments reinforce photo editing behavior.

Future research might investigate the outcome of photo editing behavior in contexts like dating platforms. As especially adolescents are vulnerable in terms of self-esteem and appearance-based self-worth, further research should also be conducted on the impact of photo editing behavior on this vulnerable target group. Future research might also explore more systematically reasons why users of SNSs edit their selfies and what motivates them to engage in photo editing behavior.

## Electronic supplementary material

Below is the link to the electronic supplementary material.

Supplementary Material 1

Supplementary Material 2

Supplementary Material 3

## Data Availability

The datasets and materials used and/or analysed during the current study are available online at: https://osf.io/kz3gb/?view_only=02591d9f59544570853fa7d394c2bfc5.

## Footnotes

1 The hypotheses were not preregistered.
