# Supplementary material for: How photo editing in social media shapes self-perceived attractiveness and self-esteem via self-objectification and physical appearance comparisons
Source: BMC Psychol. 2023 Apr 6;11:99. doi: 10.1186/s40359-023-01143-0 (PMC10080933; doi:10.1186/s40359-023-01143-0)
Supplement: Supplementary file 1 — Supplementary Material 1 [file 40359_2023_1143_MOESM1_ESM.pdf]

APPENDIX C: INTERCORRELATIONS

	(1)	(2)	(3)	(4)	(5)	(6)	(7)	(8)	(9)	(10)
(1) IAQ	-									
(2) Active	.921**	-								
(3) Passive	.586**	.373**	-							
(4) PES	.358**	.207**	.223**	-						
(5) SOBBS	.286**	.223**	.219**	.221**	-					
(6) SOBBS: F1	.298**	.213**	.256**	.227**	.931**	-				
(7) SOBBS: F2	.176**	.168**	.099**	.124**	.809**	.562**	-			
(8) PACS	.264**	.174**	.222**	.238**	.722**	.717**	.533**	-		
(9) BES: A	-.127**	-.069 ns.	-.135**	-.146**	-.599**	-.569**	-.477**	-.536**	-	
(10) RSES	-.081ns.	-.044 ns.	-.092*	-.137**	-.455**	-.390**	-.423**	-.368**	.604**	-

Note. Df = 401, IAQ = Instagram Activity Questionnaire, PES = Photo-Editing Scale, SOBBS = Self-Objectification Beliefs and Behaviors Scale, PACS = Physical Appearance Comparison Scale, BES: A = Body Esteem Scale: Appearance, RSES = Rosenberg Self-Esteem Scale
