# Supplementary material for: How photo editing in social media shapes self-perceived attractiveness and self-esteem via self-objectification and physical appearance comparisons
Source: BMC Psychol. 2023 Apr 6;11:99. doi: 10.1186/s40359-023-01143-0 (PMC10080933; doi:10.1186/s40359-023-01143-0)
Supplement: Supplementary file 2 — Supplementary Material 2 [file 40359_2023_1143_MOESM2_ESM.docx]

**Appendix B – Back-translation procedure of the BES subscale Appearance**

| Appearance Scale of the BES | | | |
| --- | --- | --- | --- |
|  | Item | Translation | Back-Translation |
| 1 | I wish I looked like someone else. | Ich wünschte, ich würde wie jemand anderes aussehen. | I wish I looked like someone else. |
| 2 | There are lots of things I’d change about my looks if I could. | Es gibt viele Dinge, die ich an meinem Aussehen ändern würde, wenn ich könnte. | There are a lot of things about my appearance that I would change if I could. |
| 3 | I wish I looked better. | Ich wünschte, ich würde besser aussehen. | I wish I would look better. |
| 4 | I worry about the way I look. | Ich mache mir Sorgen um mein Aussehen. | I worry about the way I look. |
| 5 | My looks upset me. | Mein Aussehen stört mich. | The way I look bothers me. |
| 6 | I look as nice as I’d like to. | Ich sehe genauso gut aus, wie ich gerne aussehen würde. | I look just as good as I would like to look. |
