# Supplementary material for: How photo editing in social media shapes self-perceived attractiveness and self-esteem via self-objectification and physical appearance comparisons
Source: BMC Psychol. 2023 Apr 6;11:99. doi: 10.1186/s40359-023-01143-0 (PMC10080933; doi:10.1186/s40359-023-01143-0)
Supplement: Supplementary file 3 — Supplementary Material 3 [file 40359_2023_1143_MOESM3_ESM.docx]

**Appendix A – Back-translation procedure of the SOBBS**

| Self-Objectification Beliefs and Behaviors Scale (SOBBS) | | | |
| --- | --- | --- | --- |
|  | Item | Translation | Back-Translation |
| 1 | Looking attractive to others is more important to me than being happy with who I am inside. | In den Augen anderer attraktiv auszusehen, ist mir wichtiger als mit meinen inneren Werten zufrieden zu sein. | Looking attractive in the eyes of other people is more important to me than being satisfied with my inner values. |
| 2 | I try to imagine what my body looks like to others (i.e., like I am looking at myself from the outside). | Ich versuche mir vorzustellen, wie mein Körper auf andere wirkt (z. B. so als ob ich mich von außen betrachten würde). | I try to imagine how other people perceive my body (e.g. as if I were looking at myself from an outside perspective). |
| 3 | How I look is more important to me than how I think or feel. | Wie ich aussehe, ist mir wichtiger als das, was ich denke oder fühle. | The way I look is more important to me than my thoughts and feelings. |
| 4 | I choose specific clothing or accessories based on how they make my body appear to others. | Ich wähle meine Kleidung oder Accessoires danach aus, wie sie meinen Körper auf andere wirken lassen. | I choose my clothes and accessories based on how they make my body appear to others. |
| 5 | My physical appearance is more important than my personality. | Mein Aussehen ist wichtiger als meine Persönlichkeit. | The way I look is more important than my personality. |
| 6 | When I look in the mirror, I notice areas of my appearance that I think others will view critically. | Wenn ich in den Spiegel schaue, fallen mir Bereiche meines Aussehens auf, von denen ich denke, dass sie von anderen kritisch betrachtet werden. | When I look in the mirror, I notice areas of my appearance that I think others look at critically. |
| 7 | I consider how my body will look to others in the clothing I am wearing. | Ich überlege, wie mein Körper in der Kleidung, die ich trage, aus der Sicht anderer aussieht. | I think about how my body is perceived by others based on the clothes that I wear. |
| 8 | I often think about how my body must look to others. | Ich denke oft darüber nach, wie mein Körper auf andere wirken muss. | I think a lot about how my body is perceived by others. |
| 9 | My physical appearance says more about who I am than my intellect. | Mein Aussehen sagt mehr darüber aus, wer ich bin als mein Intellekt. | The way I look says more about who I am than my intellect. |
| 10 | How sexually attractive others find me says something about who I am as a person. | Wie sexuell attraktiv mich andere finden, sagt etwas darüber aus, wer ich als Person bin. | How sexually attractive other people find me says something about who I am as a person. |
| 11 | My physical appearance is more important than my physical abilities. | Meine körperliche Erscheinung ist wichtiger als meine körperliche Verfassung. | My physical appearance is more important than my physical abilities. |
| 12 | I try to anticipate others’ reactions to my physical appearance. | Ich versuche, die Reaktionen anderer auf mein Aussehen einzuschätzen. | I try to anticipate how people could react to the way I look. |
| 13 | My body is what gives me value to other people. | Durch meinen Körper bin ich für andere Menschen wertvoll. | I am valued by other people because of my body. |
| 14 | I have thoughts about how my body looks to others even when I am alone. | Auch wenn ich allein bin, mache ich mir Gedanken darüber, wie mein Körper auf andere wirkt. | Even when I am alone I think about how my body could be perceived by others. |
